# Coherent multiple scattering in small-angle scattering experiments: modeling approximations based on the Born expansion

**DOI:** 10.1107/S1600576725006685

**Published:** 2025-09-04

**Authors:** Henrich Frielinghaus, Cedric J. Gommes

**Affiliations:** a Forschungszentrum Jülich GmbH, Jülich Centre for Neutron Science JCNS at MLZ, 85748 Garching, Germany; b Department of Chemical Engineering, University of Liège B6 A, 3 Allée du Six Août, 4000 Liège, Belgium; Australian Centre for Neutron Scattering, ANSTO, Australia

**Keywords:** coherent multiple scattering, Mie scattering, USANS, ultra-small-angle neutron scattering, USAXS, ultra-small-angle X-ray scattering

## Abstract

We start from an analytical formulation for the coherent multiple scattering treatment – similar to Mie scattering – for spherical particles. Then, we revisit the Born approximation with an approximation for all higher-order terms. Finally, we draw conclusions from those calculations and formulate an approximative model to describe ultra-small-angle neutron scattering and ultra-small-angle X-ray scattering data. In all calculations, we can specify the conditions for coherent multiple scattering. Several examples are provided to show the quality of the simple approximation in comparison with exact calculations and experiments.

## Abstract 2

A simple approximative approach is presented to model ultra small-angle neutron scattering and ultra small-angle X-ray scattering data when coherent multiple scattering is present. The underlying principles, exact solutions – similar to Mie scattering – and approximations are presented.

## 1. Introduction

In elementary discussions of small-angle scattering of neutrons or X-rays (SANS or SAXS), it is generally assumed that the particle interacts with the sample through a single scattering event. This assumption underlies the Born approximation, whereby the scattered intensity is analyzed in terms of the correlation function of the scattering-length density. This formalism, however, breaks down for large scattering cross sections or thick samples. In that case, multiple scattering has to be considered.

In the historical approach of Schelten & Schmatz (1980 ▸), the successive scattering events undergone by a particle are considered to be independent of one another. This is justified if the following two conditions are satisfied. First, the consecutive scattering events have to occur far enough from each other for the local structures at scattering points to be uncorrelated such that the particle coherent length is smaller than the mean free path of the probe. Second, the sample thickness must be large enough to cover multiple scattering events. Then, the scattering patterns are just added up independently. Many reports have been published to describe these effects and to propose deconvolution procedures for data analysis (Jaksch *et al.*, 2021 ▸; Jensen & Barker, 2018 ▸; Copley, 1988 ▸; Ji *et al.*, 2022 ▸). We refer to this situation as incoherent multiple scattering, which is not to be confused with incoherent neutron scattering by hydrogen atoms.

When the scattering events come closer to each other, the underlying structures in the sample are correlated, and coherent superposition of different waves may occur (Mazumder & Sequeira, 1992 ▸). This effect is captured by the characteristic length scale ξ of the sample, which most experimentalists consider to be the correlation length relevant to the first-order Born approximation (Roe, 2000 ▸; Hamley, 2021 ▸). The validity of the Born approximation means that only one scattering event happens within the sample.

The coherent superposition of different waves is already well established in theoretical concepts of reflectivity measurements (Gibaud, 1999 ▸), the dynamic treatment of single-crystal diffraction (Authier, 2001 ▸) and small-angle light scattering from independent particles (Mie, 1908 ▸). The complexity of the theories increases as one considers more adaptations to the sample structure as indicated in this list. In the matrix formalism of reflectivity (Gibaud, 1999 ▸), only the normal direction of the sample with resulting reflections from there is considered. The distorted wave Born approximation (Pospelov *et al.*, 2020 ▸) – in terms of multiple scattering – does not go beyond this understanding. For single-crystal scattering (Authier, 2001 ▸), all three dimensions are involved. For the well known Mie scattering by spherical particles (Mie, 1908 ▸), the complex boundary conditions of the whole surface are taken into account. Although this was derived for polarized light, a simplified version is available for quantum scalar waves. The latter is applicable to neutron scattering, as well as to light scattering when polarization is not an issue.

The conditions for multiple scattering to occur are governed by the interplay of various parameters, namely the wavelength λ of the incoming particle, a characteristic size ξ of the scattering microstructure and the scattering contrast within the sample Δρ.

In a simplified qualitative understanding, the sample must contain large enough inner surfaces where reflections can occur. That brings us automatically to the domain of very small angle and ultra-small-angle scattering (VSAS and USAS) (Barker *et al.*, 2005 ▸; Magerl *et al.*, 2024 ▸; Ji *et al.*, 2022 ▸; Zhang & Ilavsky, 2010 ▸), where we would locate the effect of coherent multiple scattering. A certain treatment (Hentschel *et al.*, 1987 ▸) allows one to describe a 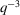 power-law ‘scattering’ pattern for fibers which orientationally averaged would agree with the well known 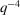 Porod scattering. Here, we formulate an approximation within which we describe deviating scattering patterns that differ from the Born approximation. This is a matter also of coherence from the probe: does the coherence volume of the probe contain the correlation volume of the sample or not? If it does, waves from different interface spots can superimpose coherently; if not, we observe independently refracted beams. In this article we derive expressions for the coherent multiple scattering and connect the findings to already well known observations. In this way, we hope to extend the understanding and interpretation of small-angle scattering experiments.

## 2. Exact solution for a spherical colloid

The elastic scattering cross section of a sphere in the first-order Born approximation is a central result in small-angle scattering (Roe, 2000 ▸; Hamley, 2021 ▸). It is given by the following well known expression: 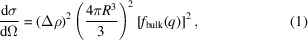
where *R* is the radius of the sphere, 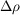 is its scattering-length density contrast with respect to the surrounding solvent (a full list of all symbols is given in Table 1 ▸) and 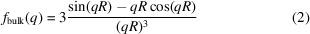
is the Fourier transform of the sphere (Pedersen, 1997 ▸), normalized such that 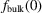 is unity. The volume of the sphere is 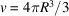. Here *q* is the modulus of the scattering wavevector defined such that 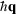 is the momentum difference between the incoming and scattered neutrons. The notation ‘bulk’ highlights that the quantity relates to the volume of the sphere, to differentiate it from the surface scattering which we introduce later.

Introductory texts often overlook that equation (1) is not an exact result of small-angle scattering. It is an approximation that holds in the limit of small contrast, as we discuss in detail in Section 3. The exact scattering cross section for an incoming particle with energy *E* is obtained by solving the Schrödinger equation (Squires, 1996 ▸), which can be written as
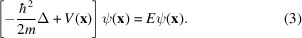
Δ is here the Laplace operator, which accounts for the momentum contribution to the Hamiltonian, with *m* being the mass of the particle. The second contribution is the structure-dependent potential 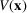 which describes the interaction of the particle with the sample. In the case of neutrons, the interaction is usually with the nuclei via strong nuclear forces. Moreover, in the typical conditions of small-angle scattering, the neutron wavelength is generally much larger than inter­atomic distances, so the Fermi pseudopotential can be applied. In that case, the potential is proportional to the local scattering-length density, namely 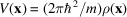 (Squires, 1996 ▸).

If the energy *E* is larger than the potential, the incoming wave is not bound by the sample interaction. In this case, the solution of the Schrödinger equation takes the form of a scattered wave,
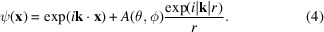
The first term describes the incoming wave with a momentum vector 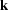, related to the energy via 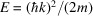. The second term is the scattered wave observed far away from the sample such that the near-field is neglected (equivalent to the Fraunhofer versus Fresnel condition in light scattering). It is implicit in equation (4) that the scattering process is elastic, *i.e.* that there is no energy transfer between the neutron and the colloid. This is a consequence of the potential 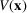 being independent of time, which is an approximation because all samples are subject to thermal motion. The conditions for elastic scattering, however, are reasonably satisfied if the colloid is moving much slower than the neutron (Monkenbusch & Richter, 2007 ▸). With all these caveats in mind, the elastic scattering cross section is related to the scattering amplitude *A* via 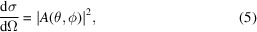
where the angular dependence is on θ only in the case of isotropic samples.

The potential 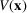 can be as complicated as any realistic microstructure of material can be. Exact analytical expressions for the scattering amplitude are available only for very simple potentials. Classical examples include the Yukawa and Coulomb potentials (Tong, 2017 ▸; Cohen-Tannoudji *et al.*, 1986 ▸; Chong, 2024 ▸), which are used in high-energy physics but are not directly relevant to small-angle scattering. Here, we consider scattering by a single spherical colloid with sharp interfaces. In other words, we assume 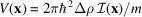 with the indicator function 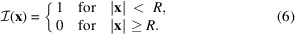
Taking the boundary conditions for the quantum-mechanical waves into account (Cohen-Tannoudji *et al.*, 1986 ▸; Chong, 2024 ▸), one can derive the following formula: 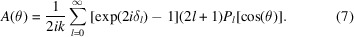
Here we used the Legendre polynomials 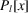. For the phases 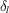 we obtain the following expression: 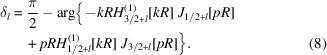
Note that equation (8) is expressed in terms of the commonly known Bessel function 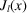 and the Hankel function of the first kind 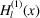, while other normalizations are often used in the literature of quantum mechanics (Cohen-Tannoudji *et al.*, 1986 ▸; Chong, 2024 ▸). For the phase 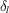, the exact normalizations of 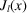 and 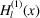 do not play a role. We also included simplifications for the derivatives of both functions.

This way of dealing with multiple scattering has an alternative formulation given by Berk & Hardman-Rhyne (1986 ▸) using an integral form that was already developed as a Wentzel–Kramers–Brillouin approximaton by Weiss (1951 ▸). The magnitude *p* is the momentum inside the colloid, which is connected to the momentum *k* according to
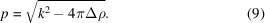
This formula is also well known in reflectometry experiments (Gibaud, 1999 ▸). For differing momenta inside and outside the colloid, together with the continuity of the wavefields at the boundary, many formulations for multiple scattering become equivalent, be it for light (Mie, 1908 ▸; Olaofe, 1970 ▸) or acoustic waves (Faran, 1951 ▸). Apart from that, the contrast 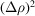 needs to be replaced by 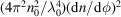 (Daicic *et al.*, 1995 ▸) for light. However, at present, we do not want to comment any further on static light scattering results.

**Figure 1:**
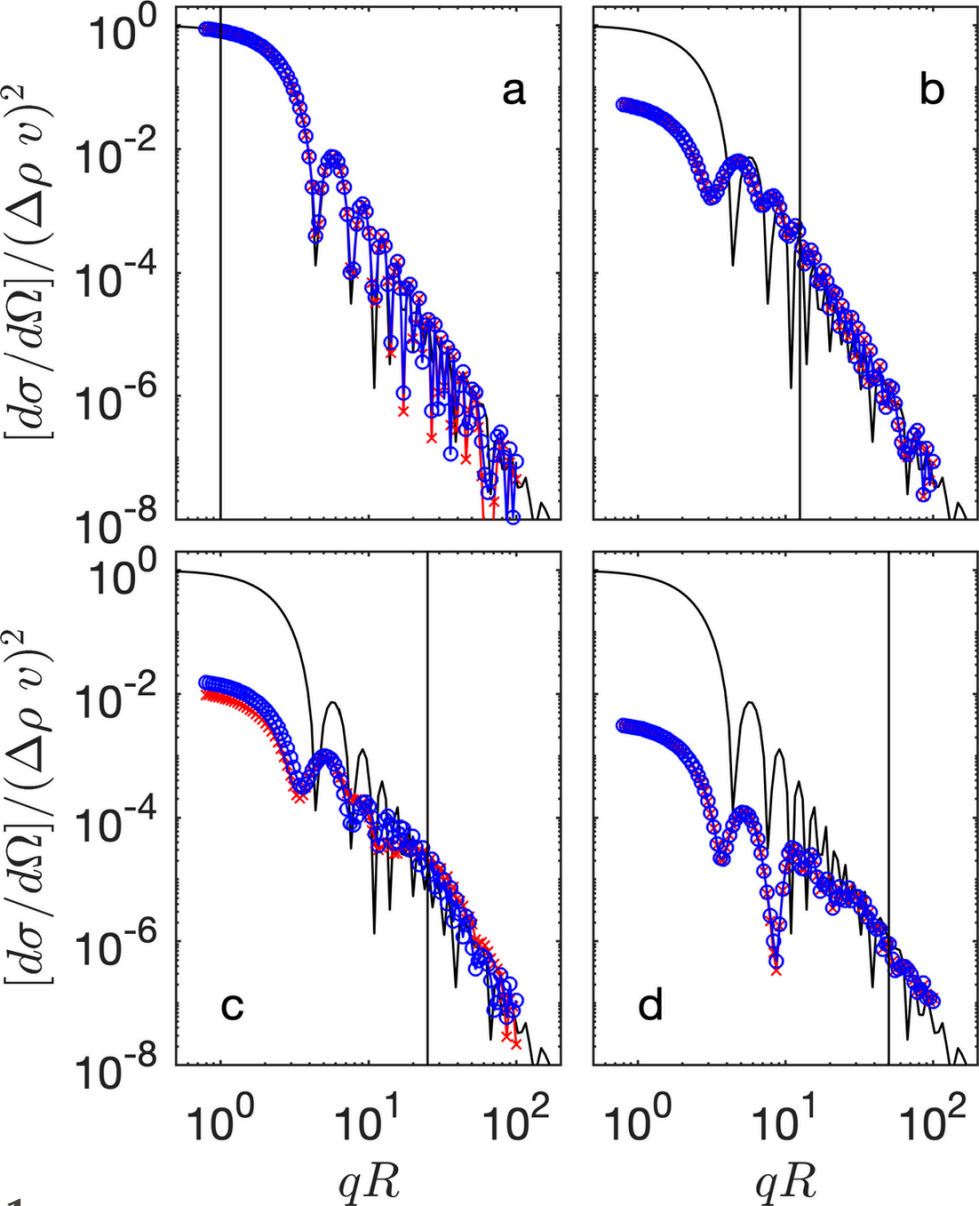
Exact scattering cross sections of a sphere calculated from equation (7) normalized to contrast and volume with (*a*) 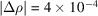, *R* = 100 (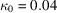); (*b*) 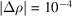, *R* = 5000 (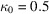); (*c*) 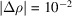, *R* = 100 (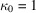) and (*d*) 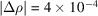, *R* = 5000 (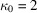). In all cases *k* = 1. The red symbols are for 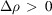 and the blue symbols for 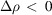. The solid black line is the form factor from equation (1), and the vertical black line is an estimation for the transition to bulk scattering which we discuss at a later stage (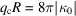). This transition is also supported by the work of Berk & Hardman-Rhyne (1986 ▸).

The formulae in equations (7)–(8) result from the exact quantum-mechanical treatment, and they therefore include the coherent multiple scattering effects. The corresponding scattering cross sections are plotted in Fig. 1 ▸. In order to facilitate the comparison with small-angle scattering, the latter are plotted not against the angle θ but against the momentum transfer 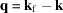, where 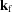 is the final momentum, after scattering. The relation to the scattering angle is 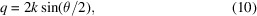
which is classical in small-angle scattering.

**Figure 2:**
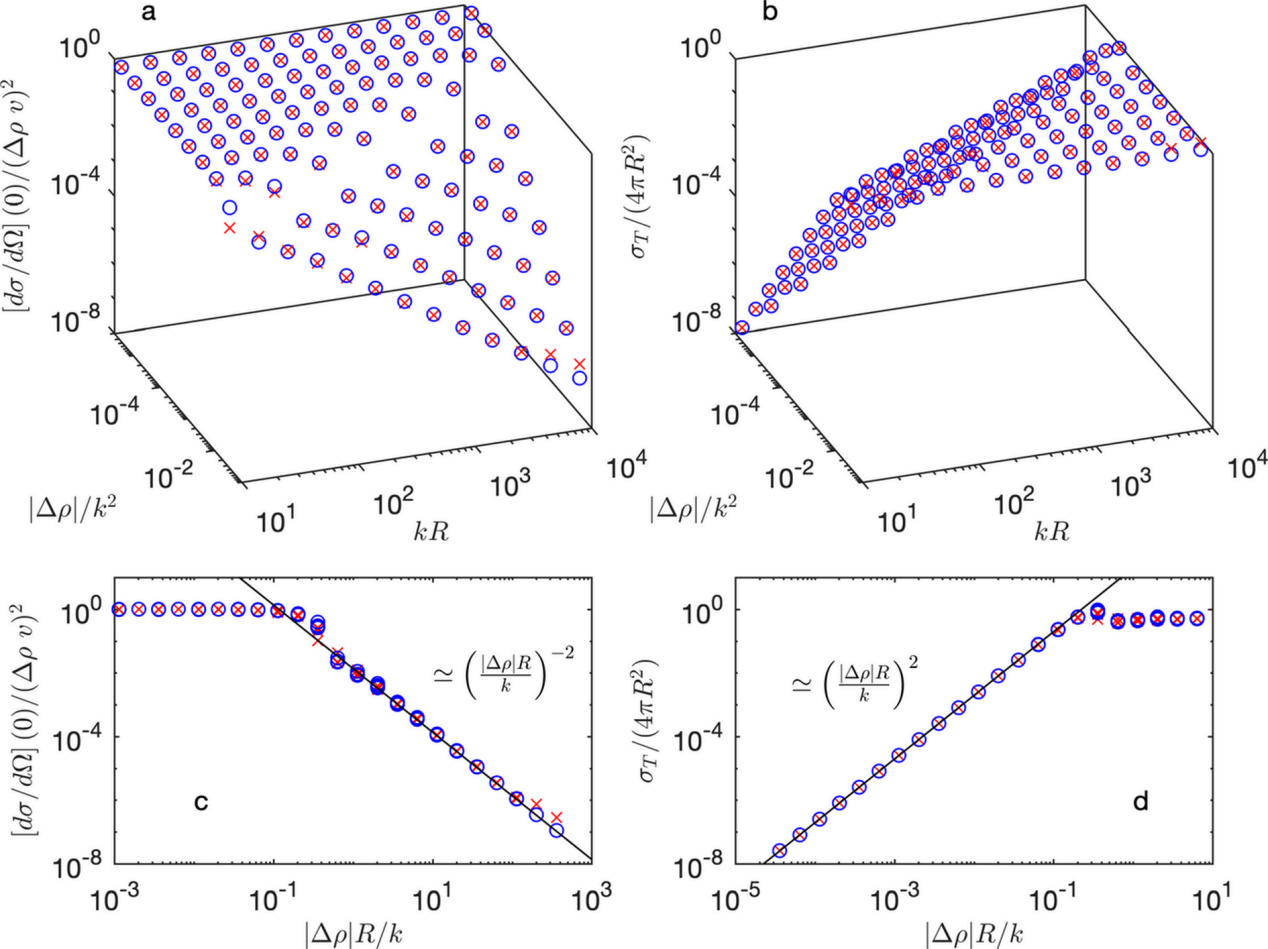
Exact value of the forward (*a*) and total (*b*) scattering cross sections, as a function of the dimensionless parameter 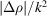 and *kR*, and the same quantities as a function of 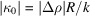. The red symbols are for 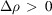 and the blue symbols for 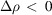. The solid black lines in (*c*) and (*d*) are empirical fits.

In order to synthetically analyze the various scattering regimes, Fig. 2 ▸ displays the forward scattering cross section, estimated from equation (7) as 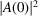, and the total scattering cross section 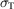. The latter quantity is an integral of 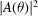 over all the directions on the unit sphere. Thanks to the optical theorem of scattering theory (Tong, 2017 ▸), however, it can be obtained from the imaginary part of the forward scattering amplitude 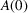 as 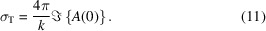
In all generality, given the dimensions of the three physical parameters that control the scattering – *k* (Å^−1^), 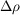 (Å^−2^) and *R* (Å) – their effect is captured by just two dimensionless numbers, which in the figure were chosen to be 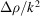 and *kR*.

Although two dimensionless parameters are, in principle, necessary to describe the scattering, it appears empirically from Fig. 2 ▸ that the main characteristics are captured by a single number. That number can be identified by noting that the occurrence of multiple scattering is necessarily controlled by the contrast 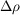, and that its mathematical dependence in equation (8) is exclusively through the combination *pR*. From equation (9), the approximate relation is 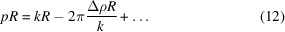
for small contrasts. To the leading order, it therefore appears that the influence of parameters *k*, *R* and 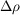 is through their dimensionless combination 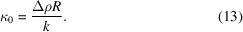
As shown in Figs. 2 ▸(*c*) and 2 ▸(*d*), the dimensionless number 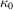 indeed captures the main characteristics of the scattering by a spherical colloid. In the literature (Berk & Hardman-Rhyne, 1986 ▸), a similar parameter 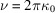 has been discussed in the same context.

To sum up the findings from Fig. 2 ▸, the transition from single to coherent multiple scattering occurs at 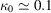 in the case of a sphere. For lower 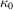, the scattering is well described by the first-order Born approximation. In particular, the forward and total scattering both scale with the squared volume of the particle, and with the squared scattering contrast. For larger 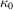, the total scattering cross section reaches the value 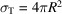 corresponding to the exact scattering cross section of a quantum-mechanical hard sphere (Tong, 2017 ▸; Chong, 2024 ▸). In that regime, the forward scattering is independent of the contrast and it scales with 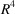, *i.e.* with the squared area of the particle. The scattering pattern then describes the projected shadow of the colloid, *i.e.* a circular disc (Weiss, 1951 ▸).

## 3. Generalization to arbitrary structures

### 3.1. The Born series and first-order approximation

To discuss scattering in general terms, it is convenient to introduce the indicator function of the colloid phase 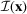, which is equal to 1 if 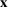 is in the colloid and to 0 in the solvent [see equation (6)]. The space-dependent scattering-length density, in excess of the solvent, is then simply 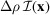, where 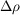 is the contrast between the solvent and colloid, as in equation (6). With this notation, a formal solution of the Schrödinger equation in equation (3) is provided by the following Lippmann–Schwinger equation (Squires, 1996 ▸):
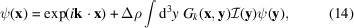
where the first and second terms are the incoming and scattered waves, respectively, similar to equation (4). In the integral, Green’s function is 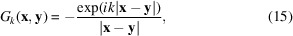
which arises from the outgoing solution of a point-like particle, *i.e.* solving 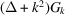 = 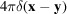.

The Lippmann–Schwinger solution in equation (14) is an integral equation, which does not provide an explicit solution of the wavefunction. One can, however, use it recursively to express the scattered wave as an infinite series, referred to as the Born series. In terms of the scattering amplitude, the solution takes the form 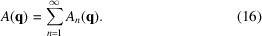
The first term in the series is obtained by approximating 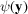 in the integral of equation (14) by the incoming wave itself 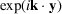. This leads to 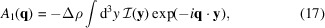
which corresponds to the first-order Born approximation.

The scattering cross section in the first-order Born approximation is then obtained through equation (5) as 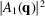. The form factor in equation (1) is the Fourier transform of this specific function. This is mathematically equal to 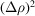 times the Fourier transform of the correlation function,
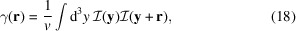
where the normalization by the volume of the colloid *v* ensures that 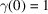. Note that 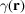 can be understood as the average value of 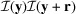 when 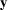 is uniformly distributed on the colloid. In the particular case of a sphere, the correlation function is 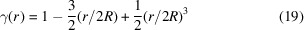
for 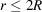 and 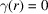 for larger distances.

The first-order Born approximation, however, ignores coherent multiple scattering effects. In general, the scattering amplitude contains an infinite series of terms, each of which accounts for a specific number of interfering scattering events. The term of order *n* takes the form 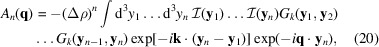
with 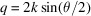 or the corresponding vectorial dependence. This is interpreted as resulting from *n* successive scatterings at points 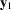 to 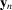, which interfere coherently to form the amplitude 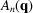. It is the latter terms that are responsible for the deviations in Fig. 1 ▸ between the exact quantum-mechanical scattering cross section and the classical small-angle scattering expression from equation (1). The interpretation of equation (20) in terms of *n* successive scattering events justifies referring to higher-order Born corrections as coherent multiple scattering.

### 3.2. The second-order Born approximation and surface scattering

**Figure 3:**
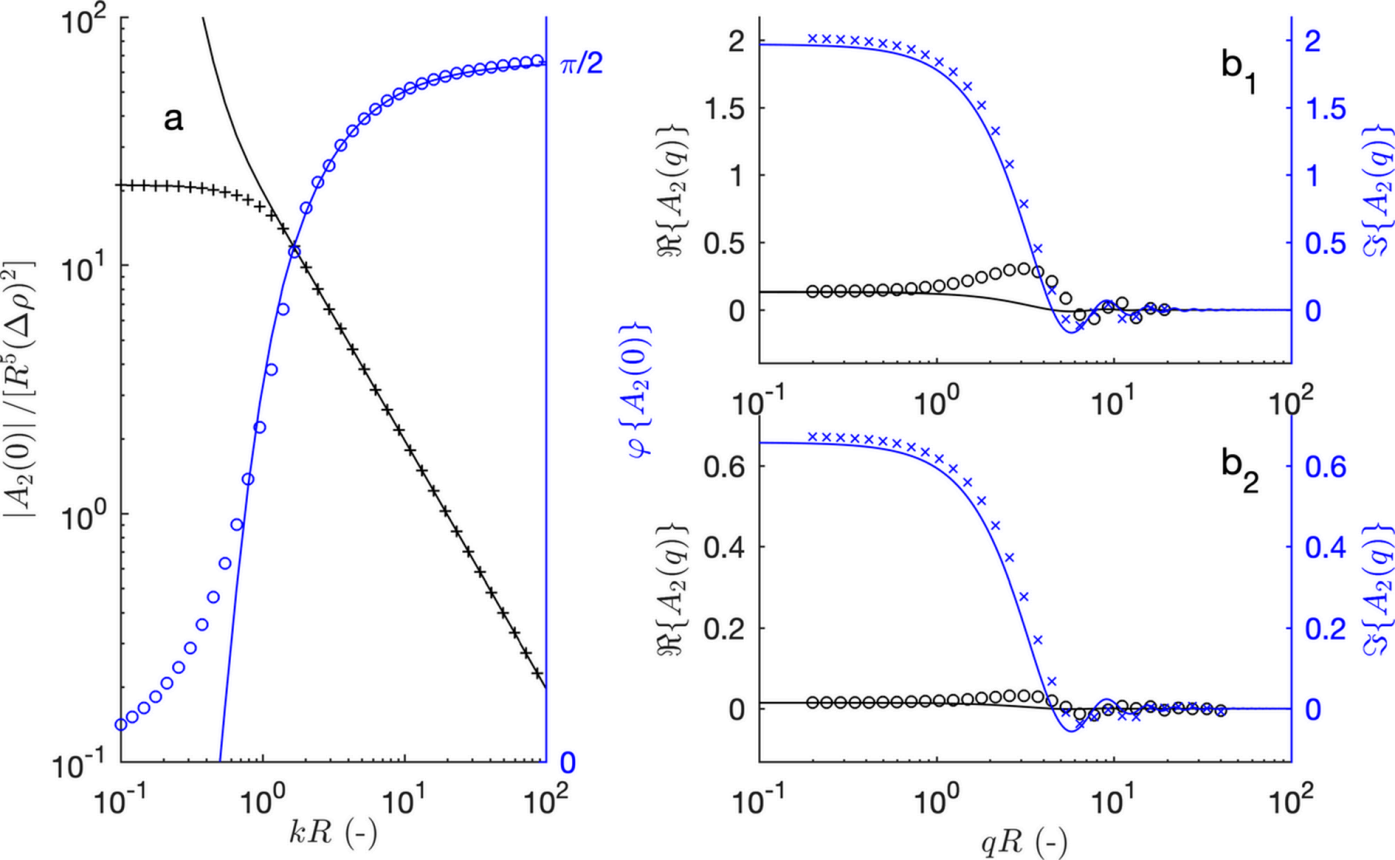
Second-order term of the Born expansion 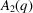 for a sphere, expressed in units of 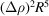. (*a*) Forward scattering amplitude 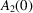 as a function of *kR*. The dots are the exact values (modulus and phase), and the solid lines are equation (29) with 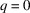. The *q*-dependent amplitudes are plotted in (*b*1) for 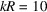 and (*b*2) for 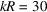 (real and imaginary parts). The dots are the exact values, calculated by evaluating numerically equation (22), and the solid lines are from equation (29).

To investigate the structural significance of the higher-order terms in the Born series, we consider here the second-order term 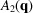, which is obtained from equation (20) for *n* = 2. Before considering its angular dependence, it is instructive to consider first the forward scattering, corresponding to *q* = 0. Without any assumption, the latter can be expressed as follows:
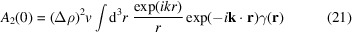
as a function of the correlation function 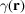 defined in equation (18). The values corresponding to a spherical particle with 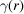 given in equation (19) are plotted in Fig. 3 ▸(*a*).

To investigate the *q* dependence of 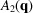, it is convenient to write it as 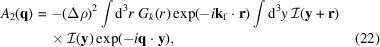
which results from equation (20) with *n* = 2, moving the Fourier integral with the highest frequency to the outermost position. In the case of small-angle scattering 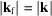 and 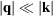. When writing the second-order term as in equation (22) the value of the high-*k* Fourier transform is determined by the small-*r* behavior of the innermost integral, *i.e.* by the structure of the surface.

The central approximation in our analysis in this Section 3.2 consists of assuming that the innermost integral in equation (22) is isotropic in 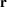. Under this isotropy assumption, we replace the integral by its rotational average, 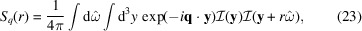
where 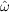 is a unit vector and its integral is over the unit sphere. Because 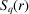 is a radial function of *r*, we can also replace the integrand of the outermost integral in equation (22) by its rotational average, namely 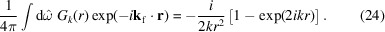
Note how the rotational average makes the distinction between 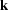 and 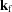 irrelevant because 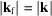 for elastic scattering. With these assumptions, the second-order Born term can be written as 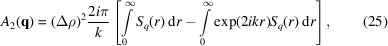
where both terms have a simple interpretation.

**Figure 4:**
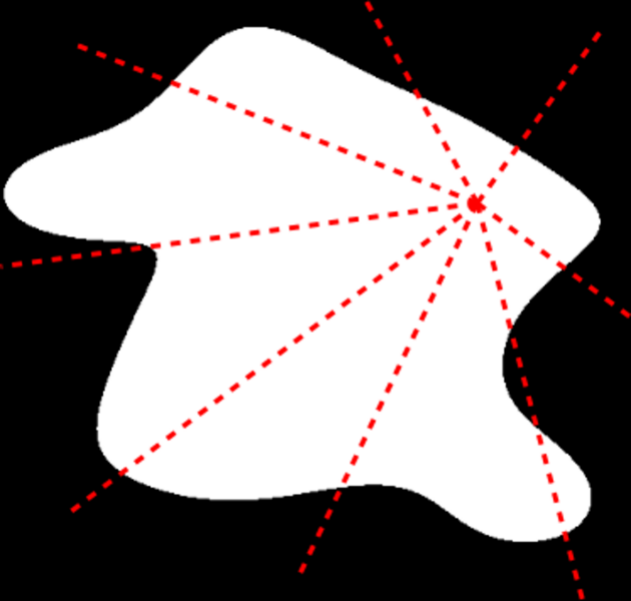
Definition of the characteristic length 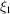 of a colloid (in white), as the average length of all radii, over all directions 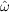 and over all possible starting points 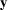 in the structure. Note that the radii may consist of several disconnected segments.

According to the definition of 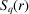 in equation (23), the first integral in the square brackets of equation (25) can be written as 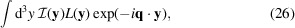
where 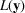 is a length, the meaning of which is illustrated in Fig. 4 ▸ (see also Appendix *A*[App appa]). Starting from any point 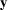 in the colloid, radii are drawn in the direction 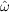, and their average length is calculated over all directions of 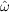. The as-defined length is space dependent, but if the dependence is weak it can be factored out from the Fourier transform and replaced by its average value 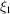, calculated over all starting points 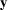 in the colloid. In other words, the first term in the square brackets of equation (25) can be approximated as 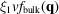.

To evaluate the second term in equation (25), we first note that in typical small-angle scattering experiments the wavelength is much shorter than the size of the colloid, so that a high-frequency approximation applies. Integrating by parts, the second integral in equation (25) can be written as 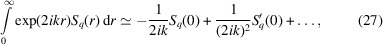
where the prime denotes a derivative with respect to *r* and the dots terms at higher reciprocal powers of *k*. From the definition of 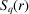 in equation (23), the dominant term is 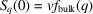 because the indicator function takes values 0 or 1, so that 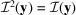. Interestingly, the derivative 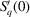 is proportional to the surface scattering 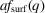, as we now explain.

**Figure 5:**
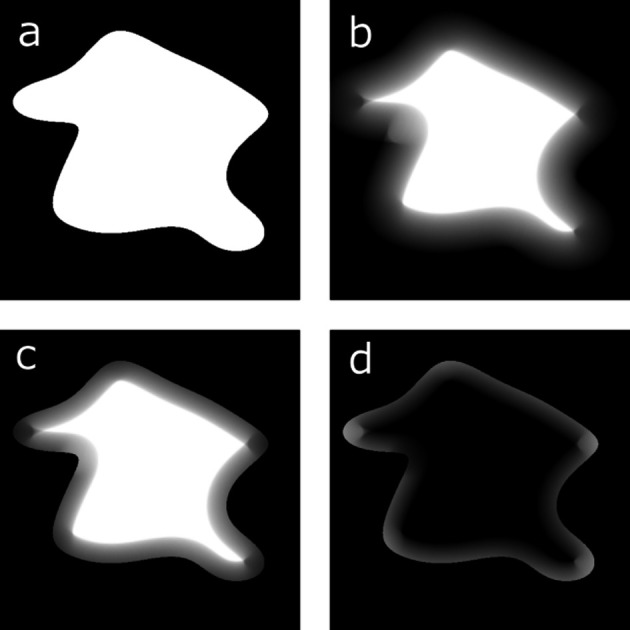
Geometrical interpretation of 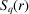 in equation (23) for small radii *r*, and origin of the surface scattering: (*a*) indicator function of the colloid 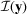; (*b*) its convolution with a small sphere 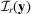; (*c*) product 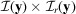; (*d*) difference between (*a*) and (*c*). The latter case describes the surface scattering that we explicitly find in equation (28).

The function 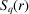 has a simple interpretation when *r* is much smaller than the size of the colloid, as relevant for equation (27) where the terms are evaluated in the limit of 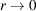. The average of 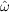 in equation (23) is equivalent to replacing the second indicator function 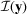 by its average value evaluated over a tiny sphere with radius *r* centered on 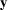, say 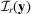 (see Appendix *B*[App appb]). This operation leaves the indicator function unchanged for all points at a distance larger than *r* from any interface. For all points closer than *r* from the surface, it replaces the sharp transition by a linear profile [see Fig. 5 ▸(*b*)]. From equation (23), 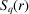 is the scattering that would result from the original colloid [Fig. 5 ▸(*a*)], from which a given *r*-dependent measure is subtracted uniformly from all over its surface [Fig. 5 ▸(*d*)]. In other words, for infinitesimally small values of *r*, one has 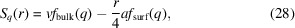
where 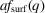 is the surface scattering amplitude, normalized in such a way that *a* is the surface area, 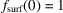, and the specific factor *r*/4 results from the integral of the linear profile sketched in Fig. 5 ▸(*d*).

Evaluating the derivative of 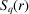 with respect to *r*, one finally gets the following general approximation for the second-order Born terms:
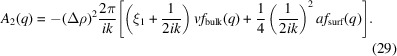
To check the validity of this approximate relation, it is compared in Fig. 3 ▸ with the direct numerical evaluation of equation (22) in the case of a spherical colloid. In that case, the bulk scattering is given by equation (2) and the surface scattering is 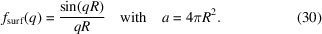
Moreover, we show in Appendix *A*[App appa] that the characteristic length is 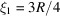 (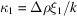) in the case of a sphere. The forward scattering, calculated from equation (29) for *q* = 0, is plotted as solid lines in Fig. 3 ▸. Deviations are observed from the exact values in the low-*kR* range, but this is irrelevant for most small-angle scattering experiments. The *q* dependence of 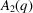 is also reasonably captured in the high-*kR* limit [see Figs. 3 ▸(*b*1) and 3 ▸(*b*2)]. Higher-order terms of the amplitudes 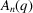 are discussed in Appendix *C*[App appc].

### 3.3. Empirical expression based on heuristic arguments

We do not attempt here to pursue the same type of analysis of the higher-order terms in the Born series as we did with the second-order term. Instead, we build on the qualitative understanding obtained so far, as well as on scaling arguments, to propose an empirical expression for scattering beyond the first-order Born approximation. We test that empirical expression against the exact quantum-mechanical solution for the sphere scattering.

To understand the general scaling of the *n*th-order Born amplitude with colloid size and *k*, the expression of 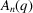 from equation (20) is conveniently rewritten as 
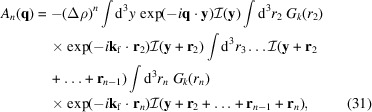
which results from reorganizing the order of the integrations. The innermost integral in equation (31) is of the type 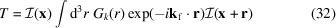
for which a few approximations can be made. First, one can note that the variable 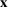 is eventually integrated many times, so that one is only interested in an average value of *T*. Furthermore, we take the average over the polar angle into account, *i.e.*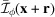 with the *z* axis along the incoming beam, *i.e.* parallel to 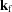. For the remaining azimuthal angle we still keep the integration.

At this point we change the argumentation and go back to the integration over the space of 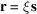 and assign to it the typical length scale ξ. For the moment we keep the value of ξ undetermined. So one gets
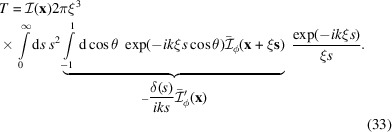
The idea behind this is that the limits of 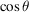 are finite, but in the limit of large 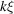 one comes close to the 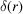 distribution. More details about the partial integration are given in Appendix *D*[App appd]. The essence of this calculation is now that we replace the derivative of 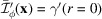 by the average correlation function. That is plausible because we consider the whole space of 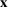. This in turn defines our preferred length scale via 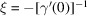. Because of the general relation between the slope of 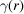 at the origin and the surface area, the length scale is simply related to the colloid volume-to-area ratio as 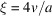 (Debye *et al.*, 1957 ▸). In the case of spheres we get 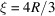. Below we will consider the different results for ξ and we define 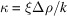. However, we obtain
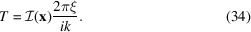
Combining the results of this section with those of Section 3.2, the overall scaling of the *n*th-order scattering amplitude is 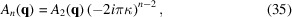
now with the new κ. With this specific dependence on *n*, the full Born expression for the forward scattering in equation (16) is a geometric series that can be easily evaluated as 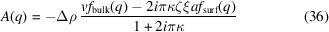
with 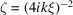.

When compared with the exact result for the sphere scattering, the analytical expression in equation (36) has some expected qualitative characteristics. In the limit of small κ, it coincides with the first Born approximation, as it should. In the limit of strong coherent multiple scattering, *i.e.* for 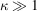, it predicts pure surface scattering, as also anticipated.

The final value for the leading term of the scattering cross section is 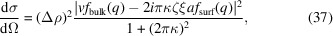
where the dimensionless number 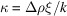 with 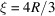 generalizes the quantity 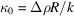 introduced when discussing the scattering by a sphere (see also Fig. 2 ▸). From the denominator 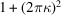, the extreme case of quantum scattering results in intransparent particles with little scattering. We discuss higher-order corrections of this equation in Appendix *C*[App appc].

**Figure 6:**
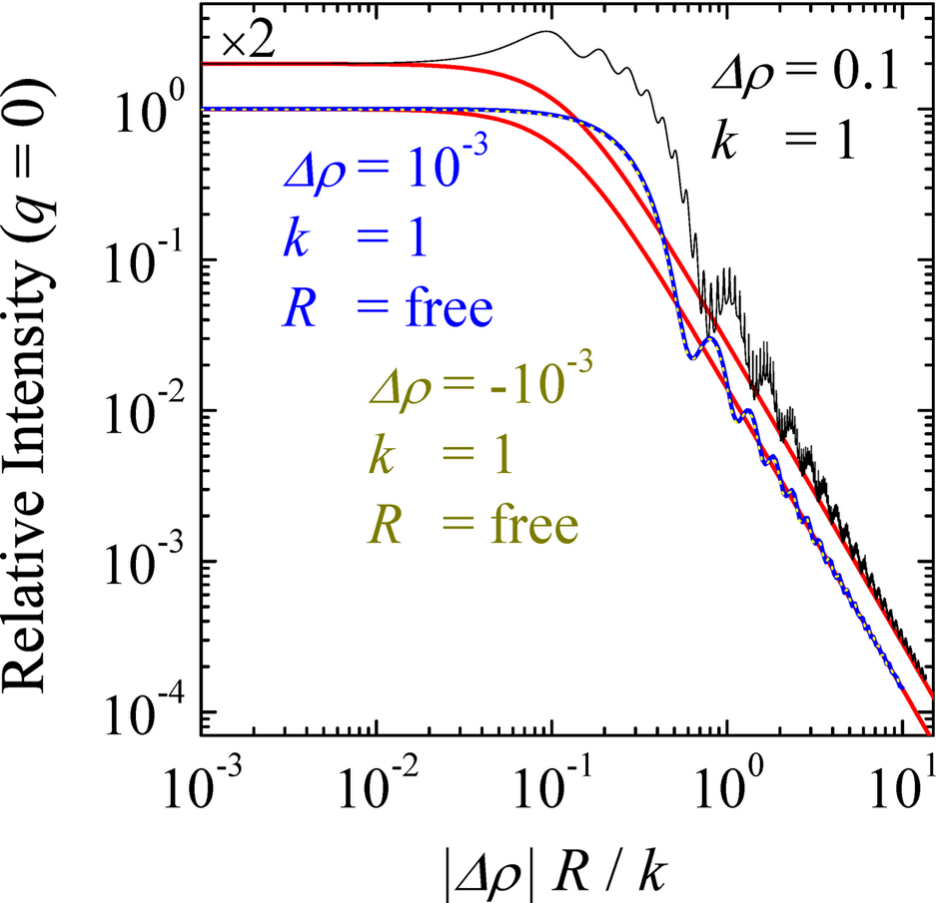
The normalized forward scattering for a spherical colloid as a function of the dimensionless parameter 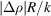. The forward scattering is normalized by the contrast and colloid volume such that it is unity in the case of no multiple scattering. The conditions are indicated in the legend (parameters are color coded). The wiggly lines present the exact calculations. The red lines represent the simplified approximation 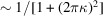.

We see that the Born series [equation (37)] correctly describes the forward scattering of a colloid including coherent multiple scattering effects. The related dominant dependence is 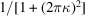 which was derived for small 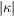 in the sense of a Taylor expansion. However, one can extend the functional dependence to 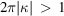 in the sense of an analytic continuation, as we see in Fig. 6 ▸. The Born series [equation (36)] also describes correctly that for large 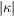 the surface scattering (as shown in Fig. 5 ▸) replaces the bulk scattering, however with a leading prefactor ζ which in our small-angle scattering approach is a small number because the correlation length ξ is assumed to be much bigger than the probe wavelength. The substitution of surface scattering for bulk scattering also becomes clear in the exact calculation of Fig. 1 ▸. From the known dependence of the forward scattering, the surface scattering must also carry the same amplitude 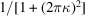 at larger 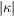 where the Born series in our approach is no longer valid. Currently, we cannot tell if our approximations are too crude or the full Born series in general is not capable of describing the transition to surface scattering correctly. Apart from the surface scattering at low *q*, we also see from Fig. 1 ▸ that the scattering profile transitions to the classical bulk scattering at 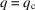, which in dimensionless units is also related to κ according to 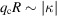. This can be interpreted as follows: on small length scales (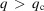), far below the correlation length ξ, the physics of the scattering process is no longer related to coherent scattering effects; this is simply the classical scattering problem of Porod scattering. This transition has already been described (Berk & Hardman-Rhyne, 1986 ▸). In some sense this corresponds to a loss of coherence when the scattering vector is only just large enough [we might assume that here ξ takes the value 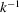 and apply this to equation (36)]. From the considerations summarized above, we now propose the following heuristic equation for the macroscopic cross section to describe the small-angle scattering profile including coherent multiple scattering for all 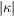: 
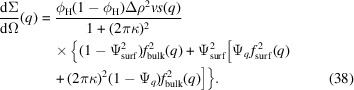
We discuss the macroscopic cross section here, which is the microscopic cross section [equation (5)] normalized to the sample volume. We now include the ideal form factors for a full sphere 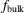 and the sphere surface 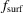 and switch between them in different cases. The first switching function 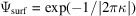 switches between coherent single scattering for small 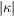 and coherent multiple scattering for large 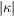. The switching within a scattering pattern from surface to bulk scattering is expressed by 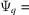

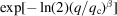
 with a characteristic scattering vector modulus 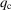 = 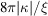 = 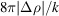 = 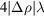. It is determined from the crossing of the unscaled surface form factor 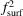 and the 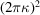 scaled bulk form factor 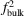. The scaling of the latter term was motivated by the argument that when the scattering vector *q* ‘observes’ the smallest structures, *i.e.* the surface only in terms of Porod scattering, the observation is not dependent on coherent multiple scattering effects anymore. For the exact crossing at 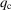, the high-*q* power law with smeared-out oscillations was taken into account only. As we will see later, the exponent β = 3.0 proved to be a useful choice. The structure factor 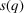 comes into play when the colloids are more concentrated such that interactions between them occur. One famous example is the Percus–Yevick structure factor (Ye *et al.*, 1996 ▸).

In principle, the scattering functions 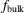 and 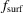 can refer to other arbitrary structures. One approach using Gaussian random fields (Gommes *et al.*, 2021 ▸) is capable of deriving expressions that can be applied to real systems. Some analytic expressions for random media with more complex formulae are derived in Appendix *E*[App appe]. The validity for different structures remains to be proved, either experimentally or theoretically.

For SANS and SAXS we know that the contrast 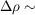 10^−5^ to 10^−6^ Å^−2^ takes rather low values and dominates the momentum difference 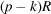. For a typical colloid size we talk about values of 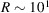 to 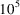
 Å (1 nm to 10 µm), typical for small-angle scattering (SAS), from VSAS to USAS. The latter might even involve larger sizes. For the momentum we talk about values of 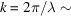 1 to 10 Å^−1^. This implies that the term 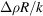 can be well below unity (for SAS) and reaches a few tens in the case of USAS experiments. For SANS and SAXS, the critical 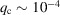
 Å^−1^ is in a rather well defined range that is typical for VSAS and USAS.

The first magnitude we want to discuss is the forward scattering, *i.e.*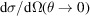 [equation (5)], which we normalize to the single scattering expectation 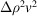 as in Fig. 1 ▸. We have displayed examples of the exact calculation [equations (7)–(8)] compared with the approximation [with the leading term 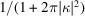 which also reflects the simplistic approach of equation (37)] in Fig. 6 ▸. The exact calculations are given by the blue, yellow and black lines. We see that the simple formula describes well the limits of single scattering 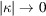 and the heavy multiple scattering 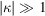. Furthermore, we can confirm that the final expression for the correlation length 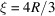 is the correct one. In the intermediate 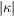 range there are deviations as follows. For the stronger contrast 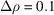 there occur two kinds of oscillations: one sharper kind of higher frequency which indicates strong resonances, and a broader kind of lower frequency which is connected to weaker resonances. The latter is maintained for the lower contrast of 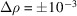 [calculations using *Maple* (https://www.maplesoft.com/) with 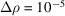 yield the same results but take an extremely long time], independent of the sign. We can state that the approximation works well after the first low-frequency oscillation (*i.e.*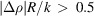) in the case of low contrasts. For this situation we expect that the surface scattering is dominant and then fully replaces the bulk scattering. Resonances may be considerable in the range 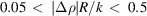 where the mixing of bulk and surface scattering may occur. However, we will also discuss this issue in more detail below and argue that the simple approach is valid in most practical cases for real samples.

**Figure 7:**
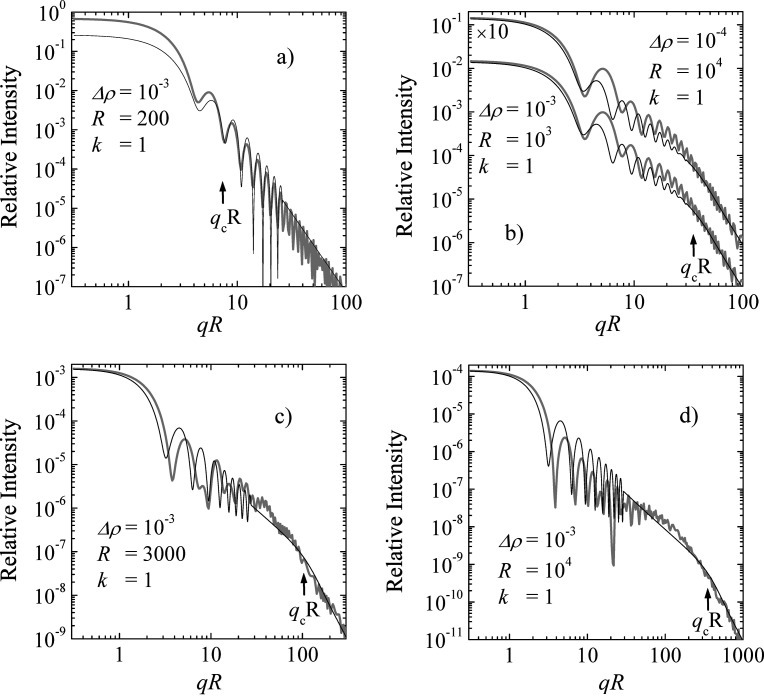
The scattering profiles for a spherical colloid under different conditions (*a*–*d*) as indicated in the legend [the natural units for 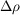 (Å^−2^), *R* (Å) and *k* (Å^−1^) are used – for faster calculations the contrast 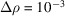 was chosen, which produces similar results to the more realistic value of 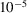 – the main variation is the parameter 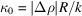 = 0.2, 1, 3 and 10 for (*a*–*d*) and 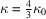]. The intensity is normalized to unity for no multiple scattering – the same as in Fig. 1 ▸. The *x* axis is normalized to dimensionless units *qR*. The gray lines represent the exact calculations. The solid lines represent the simplified approximations presented in this article [equation (38)]. For higher *q* the heavy oscillations are neglected and exchanged by the average trend. The dimensionless critical scattering vector 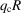 is also indicated.

We now discuss scattering patterns at different conditions (given by 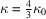) as displayed in Fig. 7 ▸. In all cases, the thicker gray curve represents the exact calculation and the thin line the simplified approximation of equation (38). High-frequency oscillations at larger *q* are omitted for the simplified calculations, for ease of visualization. We considered a range of 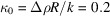 via 1 and 3 to 10. We replaced 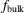 with 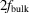 in the square brackets of equation (38) in order to represent the high-*q* end of all curves better. For all curves the parameters of 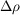, *R* and *k* are indicated. Also, the transition 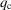 is indicated on all plots. Generally, the agreement between the approximation and the exact calculation is quite good. The high-*q* end is captured very well, and at smaller *q* there are deviations. For smaller κ the crude approximation underestimates the full theory, and the opposite is true at larger κ. The general slopes of the curves in this double logarithmic scale, when neglecting the oscillations and their possible change at 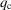, are captured quite well. The positions and amplitudes of the first fringes are captured reasonably well, but clear differences are visible and are an expression of the resonances that complicate the exact theory versus the simple approximation.

When considering real samples at rather large scales (>1 µm), there are usually wide distributions of sizes that smear out all oscillations of the scattering patterns, and so only power-law behaviors remain observable in the experiment. This averaging would also smear out the under- and overestimations of the different conditions expressed by the parameter 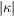. Thus, we believe that for a simple power-law scattering pattern a simple change of slopes would occur in the experiment, as described by
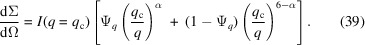
The exponent 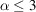 describes the surface scattering and is connected to mass fractals (the term mass is not to be confused with bulk – it expresses the real dimensionality of the surface in the 3D space). The following exponent 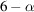 is then the corresponding structure under bulk contrast and is connected to surface fractals. The pair of exponents for 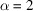 is already well described in the theory of Porod (Glatter & Kratky, 1982 ▸; Roe, 2000 ▸) for smooth surfaces of compact objects. Generally, the range for the exponent is 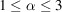 and seems to be a sharp boundary for all possible fractal structures (Martin, 1986 ▸; Kjems *et al.*, 1986 ▸). As before, we keep the description of the switching function 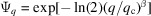. For many hierarchical structures with smeared-out fringes, the expressions derived by Beaucage (1996 ▸) give a good model function to describe small-angle data. Thus, the simple power-law expression of equation (39) can be expanded to
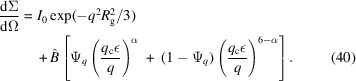
Here, the parameter 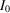 describes the forward scattering that is connected to the Guinier scattering, and the second amplitude 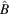 = 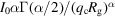 is tightly related to the remaining parameters [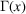 is the gamma function]. The overall size 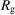 describes the appearance of the scattering surface. The second switching function is connected to 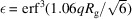. The exponent in the first switching function 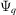 is best selected by the formula 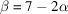 or a bigger value.

## 4. Discussion

**Figure 8:**
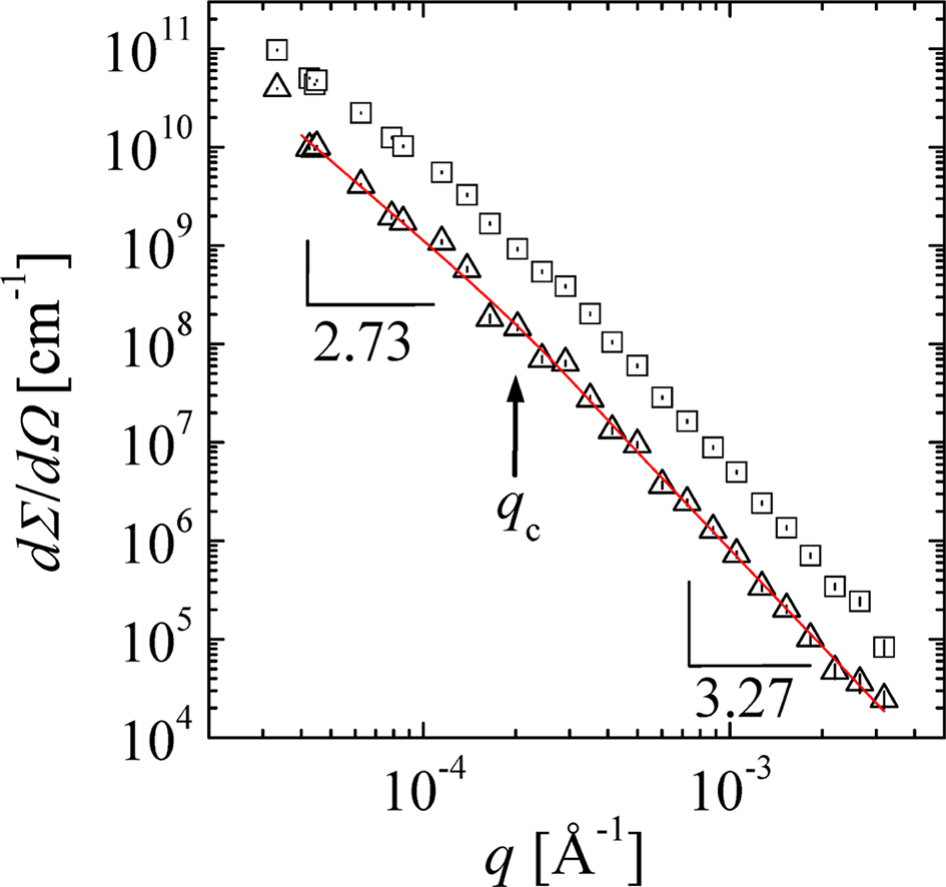
The USANS profile of a single sheet of paper (macroscopic cross section as a function of the scattering vector) from Ji *et al.* (2022 ▸). The squares indicate the original measurement that was slit desmeared. The incoherent multiple scattering deconvoluted data are indicated by the triangles. To this, the simple power-law behavior with a crossover [equation (39)] is fitted (red line, slopes are indicated). The critial scattering vector modulus 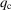 is indicated by the arrow. All error bars are plotted within the symbols.

Here, real experiments are discussed in the context of the above-mentioned formulae. A practical example was obtained by the USANS scattering of simple paper, as displayed in Fig. 8 ▸ (Ji *et al.*, 2022 ▸). The original slit desmeared data are indicated and compared with the incoherent multiple scattering corrected data. The latter data were described by the simpler formula of equation (39). The critical 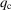 is found to be 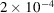
 Å^−1^, *i.e.* larger than the theoretical value. However, the spread of the two power laws of 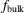 and 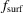 with the exponents being close to 3 is smaller, and so the crossover may be shifted to a slightly larger 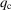. Thus, we believe that 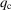 may be shifted by factors of the order 2–3 in comparison with the exact theoretical value, but this remains to be verified experimentally.

Examples in the literature may support the findings we have discussed here. For polysulfone membranes, experiments using USANS and SANS (Siddique *et al.*, 2022 ▸) display a crossover between a mass and surface fractal at 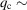 2–3 × 10^−4^ Å^−1^ which was not described any further. One other example is the already discussed scattering from paper, and the scattering of shales has been presented (Ji *et al.*, 2022 ▸). Also for silica particles with polymers in solution (Schmitt *et al.*, 2016 ▸), USAXS experiments display a crossover between mass and surface fractal behavior at 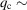 5 × 10^−4^ Å^−1^ for different silica morphologies. However, different morphologies are identified using supporting scanning electron microscopy micrographs. Thus, in the latter case, in part the different slopes could be directly interpreted in terms of the real-space structures and 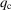 seems to be related to a real structural size. Another USAXS study found a rather low *q* ∼ 10^−4^ Å^−1^ (Munoz *et al.*, 2023 ▸) where the slopes still indicate surface fractals at lowest *q*. Here, the coherence might be insufficient if the underlying observed structures of size ξ are much bigger (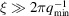). In that case, opposing surfaces from the object are not interfering in the experimental observations. A detailed analysis for the different cases is required.

**Figure 9:**
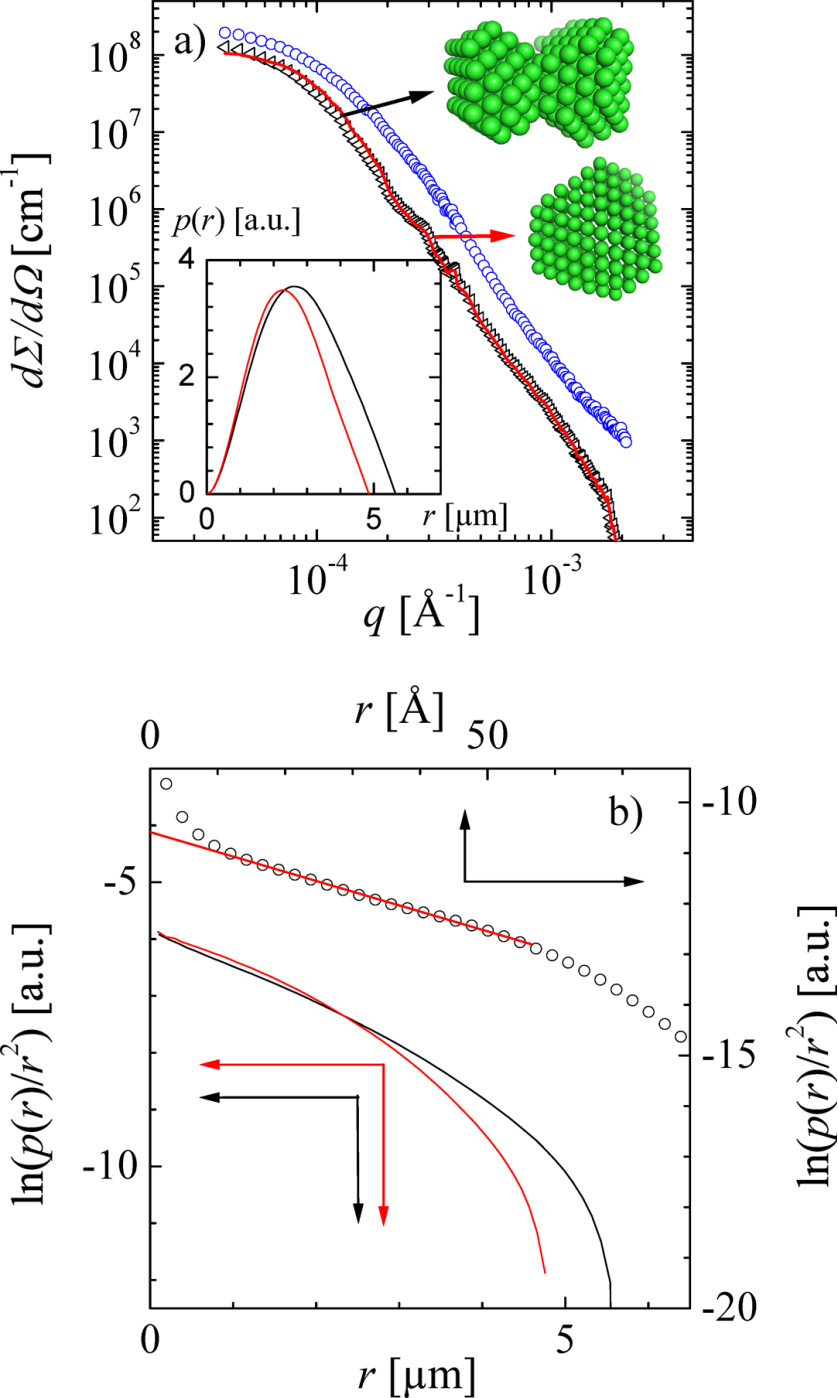
(*a*) The SANS curve of a BSA aggregate with considerable trivalent Y cations (200 mg ml^−1^ BSA and 30 mmol l^−1^ YCl_3_ at 35°C). The original measurement (blue) with incoherent scattering removed is displayed as black triangles and the corrected one as a red solid line. In the inset, the corresponding real-space correlation functions 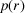 are shown. The real-space reconstructions are also added on the top right. (*b*) The logarithm of the real-space correlation function 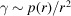 for the BSA protein aggregate before and after the correction (black and red lines, respectively). Another example from a cruciferin complex (trimers at the oil droplet interfaces of an emulsion) is also shown, where the low-*Q* upturn is due to a high-*Q* cutoff of the scattering data. Here, the line indicates the correct extrapolation to 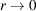.

Another example deals with a protein aggregate of bovine serum albumin (BSA) with considerable amounts of trivalent yttrium cations in D_2_O [Fig. 9 ▸(*a*)] (Soraruf *et al.*, 2014 ▸; Beck *et al.*, 2021 ▸). First, the incoherent multiple scattering is removed from the original data (blue). Then, we can see the scattering curve from the protein at this stage (black). As such, this curve does not look unusual, but when performing a real-space reconstruction using *DENFERT* (Koutsioubas & Pérez, 2013 ▸; Koutsioubas *et al.*, 2016 ▸) the structure looks slightly elongated and not isotropic anymore. From the pure uncontrolled aggregation, one would expect isotropic globular aggregates. Thus, fitting the low-*Q* part (*Q* < 0.0005 Å^−1^) with the modified Beaucage model [equation (40)] and then assuming that the high-*Q* exponent 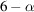 is also valid for a simple Beaucage function extending to the low-*Q* part with 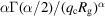 = 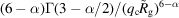 yields two functions to be solved for 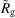. The ratio of those two model functions was applied to the real measured data, and we obtained a corrected scattering curve (red line). From this curve, we obtained a rather isotropic, globular real-space reconstruction.

As the real-space reconstruction goes hand in hand with obtaining the real-space correlation funtion 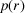 using an indirect Fourier transform algorithm (Hansen, 2000 ▸), we discuss that here as well. The original data of 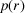 for the protein aggregates are displayed in Fig. 9 ▸(*a*), and the related real-space correlation function 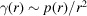 is displayed in Fig. 9 ▸(*b*). For our example, the curves look rather smooth, and one could extract the related correlation length 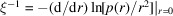 from it. In the logarithmic representation the calibration of 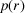 does not matter. However, when applying this method to the complex of cruciferin (trimers at the oil droplet interfaces of an emulsion) (Holderer *et al.*, 2025 ▸), we see that the low-*r* part diverges for 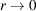. A solid red line indicates the considered low-*Q* extrapolation. The divergence is due to the high-*Q* cutoff in the original scattering data (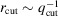). In our case, the positive background level causes the upturn at low *Q*, while an overestimated background subtraction would cause a downturn. The example shows that, if precise values for the correlation length ξ are needed, the inverse Fourier transform can provide the desired data.

For incoherent multiple scattering where the different scattering events happen independently in the sample, the criterion 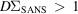 must be fulfilled. This can be calculated from an integration of the scattering pattern, *i.e.*
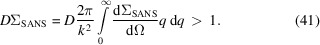
Here, we assume that the constant incoherent background was subtracted from the SANS scattering pattern. When comparing this with the criterion for coherent multiple scattering, *i.e.*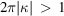, one observes the following: the case of incoherent multiple scattering appears earlier and is much more likely as long as the sample thickness *D* is larger than the structural size (*i.e.*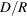 or 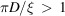). For SANS experiments this is always reasonably fulfilled, and only for surface-sensitive experiments might one observe coherent multiple scattering first (Shen & Maradudin, 1980 ▸). We refer to the magnitude 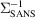 as the mean free path length.

## 5. Conclusions

We distinguish between incoherent and coherent multiple scattering based on either uncorrelated or interfering scattering events in the sample. Usually, the incoherent multiple scattering sets in first when the sample thickness is larger than structural sizes of length ξ and the mean free path length 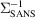. Here, the structural information is superimposed independently and this is not the focus of the current article. This effect is not to be confused with the incoherent scattering from hydrogen atoms which may or may not be a different multiple scattering effect. The coherent multiple scattering has a critical scattering vector 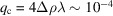
 Å^−1^ which is connected to the contrast 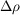 and the wavelength λ of the probe. Slight deviations of the order 2–3 of this estimation are possible. Thus, coherent multiple scattering is an issue for VSAS and USAS.

The change from incoherent to coherent multiple scattering that we describe in this article is a transitional stage mostly found for relatively weak contrasts and large structures. The ultimate stage of multiple scattering is the quantum scattering that is achieved for even larger κ and then describes the projected shadow of the structure. Thus our intermediate stage of surface scattering is an approximate description that we shed light on using a more theoretical approach, but also by discussing real SANS experiments where the mechanism can be clearly demonstrated. Thus, a rigorous treatment of our statements remains a topic for future work. As one can see in Fig. 7 ▸ there is under- and overestimation of the perfect surface scattering. This already demonstrates the transitional validity of our approach. Practically, this means that observations at much smaller *q* values may finally reveal the full quantum scattering.

For our transition from incoherent to coherent multiple scattering we further find that, as most structures of the large sizes considered here are usually polydisperse, only power laws without fringes will be observed in this range. We predict that the typical slopes below 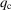 indicate mass fractals with an exponent 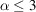. This is due to the surface scattering typical for coherent multiple scattering in this region. Above 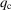 the fractal exponent transitions to 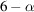, which then is the Porod scattering of the same structure. A simple formula for this effect is given by a modified Beaucage expression [equation (40)]. When new neutron instruments are developed for even smaller *q* (Magerl *et al.*, 2024 ▸), coherent multiple scattering is even more important. Only for extremely large structural sizes, *i.e.*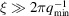 given by the smallest resolved scattering vector, does coherent multiple scattering not develop and so larger slopes 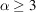 may be visible at the smallest *q* (Hentschel *et al.*, 1987 ▸) (or quantum scattering may be observed).

The recommended order of corrections to a USANS (USAXS) experiment is (1) slit desmearing and (2) desmearing of incoherent multiple scattering. After that, a stitched scattering curve including classical SANS data with many orders of length scales will display single scattering for 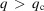 (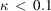) which can be interpreted as usual. At slightly higher 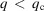 (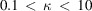), the coherent multiple scattering with the characteristic surface scattering must be taken into account. Here, the curves may be directly interpreted via equation (40) (as we did for paper scattering) or may be corrected by the ratio of equation (40) and an ideal Beaucage fit, as we demonstrated for the protein aggregates. At much smaller 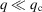 (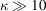), either quantum scattering may be present (with the typical shading effect) or the loss of coherence (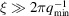) may lead to classical Porod scattering. The distinction in the latter case may be obsolete and is a topic for future work.

## Figures and Tables

**Figure 10:**
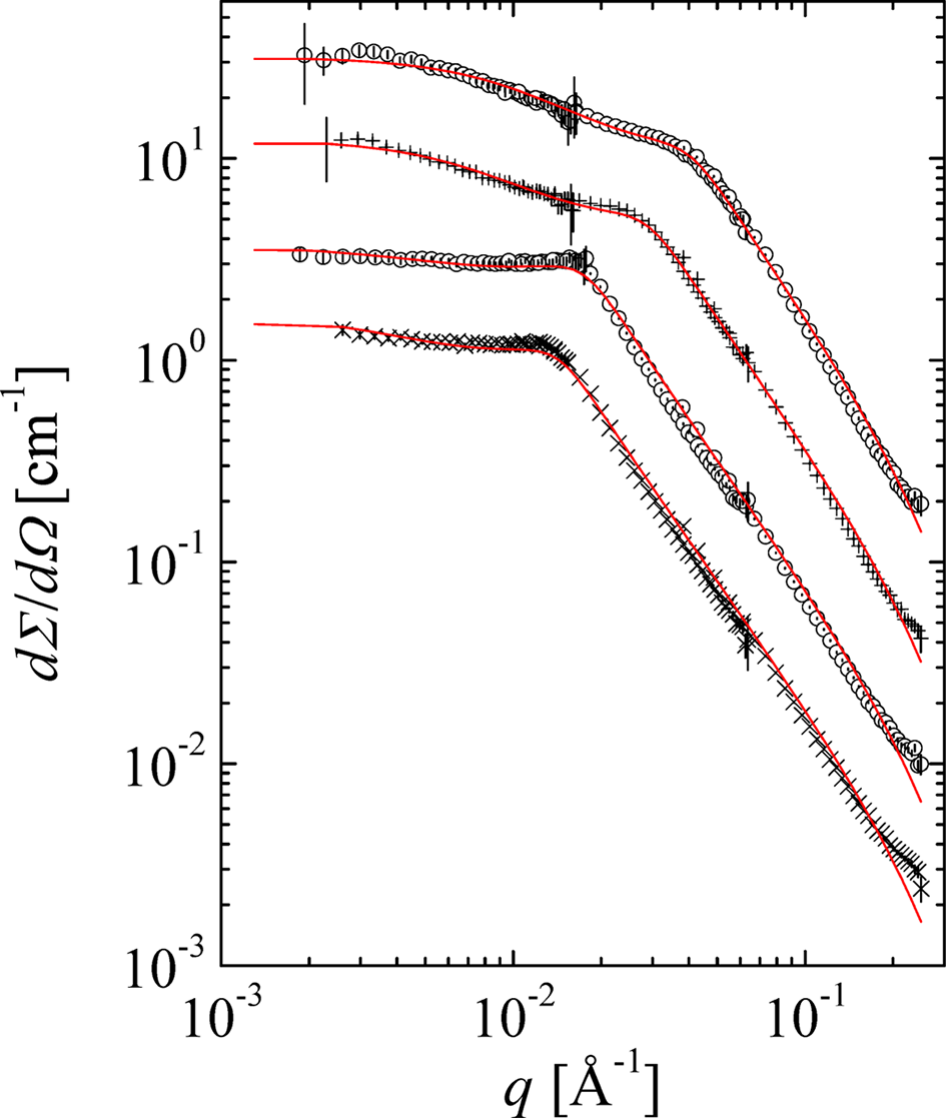
The film contrast measurements from Endo *et al.* (2001 ▸) (shifted by a factor of 0.3 each) described by equation (87) (red lines) with interpolated parameters *d* and ξ from the bulk measurements and only two free parameters 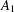 and 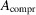.

**Table 1:** This table presents all symbols used in the article

Symbol	Meaning
*a*	Area of the colloid surface or correlation area of the surface scattering
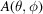	Scattering amplitude of the outgoing wave
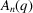	Amplitude of degree *n* from the Born series
α	Exponent of power law for surface scattering, *i.e.* fractal dimension
β	Exponent for the switching function 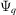
*d*	Domain spacing of a porous structure (microemulsion)
*D*	Thickness of sample
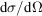	Microscopic cross section
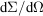	Macroscopic cross section, *i.e.* 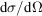 normalized to the sample volume
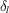	Phase function for the spherical colloid scattering
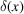	Dirac delta distribution
Δ	Laplace operator
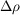	Scattering-length density difference between colloid and solvent
*E*	Quantum-mechanical energy of the probe (neutron)
ε	Switching function in the Beaucage scattering function for fractals
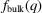	Form factor of the bulk colloid or structure
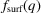	Form factor of the colloid surface or structure interface
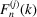	Specific scattering functions for first-order corrections
ϕ	Polar angle
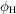	Volume fraction of hydrogenous material in the sample
*g*	Length parameter correction with 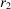 of the order 1
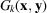	Green’s function for the scattering event
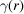	Real-space correlation function
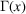	Gamma function
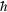	Planck constant
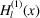	Hankel function of the first kind
*i*	Imaginary unit
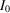	Forward scattering parameter
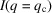	Intensity at the critical scattering vector modulus 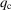
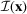	Indicator function of the colloid, *i.e.* 1 inside the colloid and 0 otherwise
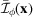	Indicator function averaged over the polar angle
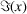	Imaginary part of the argument *x*
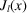	Bessel function
*k*	Modulus of the wavevector of the incoming probe (neutron), *i.e.* 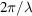
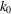	Modulus of the wavevector of the porous material structure, *i.e.* 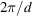
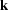	Wavevector of the incoming probe
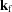	Wavevector of the outgoing probe (final momentum)
*K*	Final complex strength for coherent multiple scattering
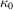	Strength of coherent multiple scattering (zeroth approximation)
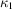	Strength of coherent multiple scattering (first approximation)
κ	Strength of coherent multiple scattering (final approach)
*l*	Index, angular quantum number
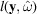	Length distribution function for certain solid angle 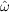 inside the colloid
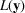	Length distribution function
λ	Probe (neutron) wavelength
*m*	Neutron mass
*n*	Index, usually connected to the order of the Born approximation
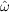	Solid angle
*p*	Wavevector inside the colloid according to the different potential *V*
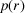	Pair distribution function
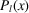	Legendre polynomial of the argument *x*
π	Circle constant
ψ	Quantum-mechanical wavefunction (non-bound state)
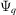	Switching function as a function of *q* between surface and classical Porod scattering
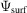	Switching function between surface and classical first-order Born approximation
*q*	Modulus of the scattering vector, *i.e.* 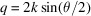
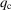	Modulus of the critical scattering vector between surface and classical Porod scattering
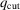	Modulus of the maximum scattering vector used to cut the *q* range of a scattering curve
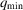	Experimental minimum scattering vector modulus
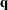	Scattering vector
*r*	Distance, *i.e.* modulus of the spatial argument 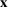 or 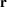
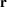	Spatial variable inside the sample
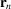	Spatial variable inside the sample with index *n*
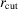	Minimum valid distance before divergence of 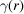 for 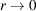
*R*	Radius of the spherical colloid
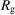	Radius of gyration from the second moment of mass distribution of the respective structure
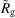	Radius of gyration for the corrected scattering function
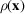	Scattering-length density profile of the sample
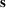	Dimensionless spatial variable, *i.e.* 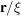
*S*	Extracted term from term *T*
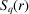	Scattering function (also for higher orders)
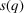	Structure factor for arrangement of several colloids
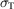	Total scattering cross section
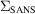	Reciprocal mean free path length given by measured SANS curve
*T*	Innermost integral of a higher-order scattering amplitude
θ	Azimuthal angle
*v*	Volume of the colloid or correlation volume of the bulk scattering
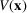	Interaction potential between probe and sample
*x*	Argument of a function (not to be confused with *r*, the modulus of 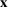 )
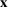	Spatial variable inside the sample
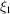	Correlation or typical length of the sample structure, first approximation
ξ	Correlation or typical length of the sample structure
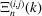	Specific scattering functions for second-order corrections
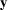	Spatial variable inside the sample
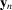	Spatial variable inside the sample with index *n*
*Z*	Dimensionless complex correlation length parameter
ζ	Dimensionless reciprocal correlation length parameter

**Table 2:** The parameters used to describe the film scattering of microemulsions (Endo *et al.*, 2001 ▸) The parameters *d* and ξ were obtained by interpolation of the bulk scattering model fitting. The only free parameters were the amplitudes 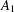 and 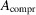.

Sample	*d* (Å)	ξ (Å)	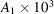 (cm^−1^ Å ^−2^)	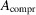 (cm^−1^)	σ (Å)
20	325	170	1.77	23.1	3.3
21	451	243	1.39	28.2	3.3
23	746	414	1.07	12.8	3.3
24	912	510	0.89 ± 0.02	27.9 ± 1.0	3.3

## Appendix A Characteristic length ξ_1_

The dominant term in the definition of the characteristic length  is the 1D integral of the correlation function, assumed to be isotropic. This definition can be generalized to non-isotropic structures as

which is equivalent to evaluating the rotational average of , before evaluating the 1D integral.

To understand the geometrical significance of , note that equation (42) is equivalent to the following three-step calculation. First, for any given point  in the colloid [ is the indicator function] and direction , the following length is defined:

as illustrated by the individual radii in Fig. 4 ▸. The position-dependent length  is defined by averaging  over all directions, namely

Finally, the characteristic length  is obtained as the average of , that is

Practically, the integral only extends over the colloid volume due to the product with . These equations result from expressing the integral operation in equation (42) in spherical coordinates, and using the general definition of the correlation function in equation (18).

In the particular case of a spherical colloid with radius *R* and centered on , the length  is

where θ is the angle between  and the direction from the origin to . Averaging over the unit sphere then provides the relation

which takes the value *R* in the center of the sphere  and  on its surface . The average value, calculated as

then provides the value  and .

## Appendix B The indicator function of the surface

To evaluate the derivative of  with respect to *r*, in the limit of , consider first the quantity

for values of *r* much smaller than any characteristic size of the scattering material. This function replaces the indicator function  by its average value calculated over a small sphere with radius *r* centered on . This operation leaves the indicator function unchanged for all points at a distance larger than *r* from any interface. For points closer than *r* from the interface, it replaces the sharp 0/1 transition by the following smooth profile:

where the distance *z* to the interface is counted negatively into the colloid and positively into the solvent. On the basis of equation (23),  is the scattering that would result from the original sample, from which a measure *r*/4 (per unit area) is subtracted all over its surface. The latter value is the integral of  in the negative *z*.

## Appendix C The higher-order amplitudes

The higher-order amplitudes can be approximated in a similar fashion as in equations (22)–(25). Thus, the general term is approximated as

Here,

where the average over all the  orientations  is implicit. The approximation in equation (51) results from assuming structural isotropy, so that each 3D integral over say  is replaced by a 1D integral over the modulus  of the rotationally averaged integrand. From the appearance of equation (52) we can see that it scales with a typical length , and finally the amplitude [equation (51)] scales with . So, the amplitude decays fast for smaller  and requires substantial corrections for large .

### c1. Zeroth-order term

To the zeroth order, the dominant term in equation (51) is

Calculating the integral of equation (52) over  provides

where  is defined in equation (44), and the second equality results from approximating  in the integral by its average value over all  in the colloid .

Repeating the same approximation on all successive integrals yields

### c2. First-order correction

The first-order corrections are

where

is the Fourier transform of  over the *j*th variable, integrated over all other variables. Integrating by parts the Fourier integral on  provides the following high-*k* approximation:

In this equation, all the unspecified variables  for  are integrated from zero to infinity, and  is the derivative of  with respect to variable . At this stage, we need to evaluate  for infinitely small values of , so that we can calculate  and . All the dots are integrals.

When evaluating , the integrals on the right side of , *i.e.* all  with , can be approximated in the same way as in equation (54). This leads to

As a particular case

where the second equality results from equation (28).

To understand how the integrals on the left of  have to be handled, *i.e.* of  with , consider first

where  has the same meaning as in equation (49). Note that we only need the behavior for small . In that limit, the innermost integral is

Note that the left-hand side is identical to the definition of  in equation (44), only with a smooth transition over a thickness proportional to . This is equivalent to removing some weight in the integral close to the interface. The constant *g* can be calculated along the same lines as in equation (50) and is approximately . Note, however, that in the case of a non-convex particle, the line in direction  crosses the interface several times. And each crossing contributes to *g* by the same amount. We will come back to this later, and we write now

To generalize this, consider

where the innermost integral is

When factoring it out of the integral, this yields a factor . All the other  integrals yield a factor . The final result is

Putting it all together, the result is

for any . And for *j* = 1, the result is in equation (60).

The final result is

### c3. Second-order correction

To be consistent, if we want to keep the surface term in , we need to keep all terms of order  in . This demands that we also consider some of the contributions to .

The second-order corrections are

where

is the Fourier transform of  over the *i*th and *j*th variables and integrated over all other variables.

We can deliberately ignore terms smaller than , so we need only consider

because the contributions proportional to the derivative of  bring an additional factor 1/*k*,

### c4. The sum of all amplitudes

We can now sum all higher-order amplitudes. However, before that we introduce the abbreviations:

and

We note that  around equation (36). Thus, the higher-order amplitude  to the second order can be abbreviated to

Note that this is identical to equation (29) for *n* = 2. For the geometric series with modifications, we can now conclude: useful sums, for any *K* (in principle with )

The complete scattering amplitude, summing all the terms, takes the form

Note that *K* is proportional to , and it is large when one enters deeply in the quantum-mechanical regime. In the limit of infinitely large *K*, the scattering amplitude is

In contrast, small *K* refers to small corrections of the simple Born approximation. This means for  we are left with only one term, namely the simple bulk scattering.

## Appendix D More details about the integral of *T*

The expression *T* results from the innermost integral of equation (31) of the higher-order scattering amplitude . In this sense it is a rescaled indicator function  or  that then gives a typical size ξ. The first step is the partial integration over the variable  in equation (33). One defines the subterm *S* according to

The first line (83) refers to the integrated term and the second line (84) includes the derivative of  which is expanded with respect to the argument . In the last line (85), we distinguish the cases for small *s* and finite *s*. In the first case, the differential quotient for the *z* component arises from (83): similarly for (84), where only even terms in  occur. The  term introduces higher orders of *s* and random phases that can all be neglected. Finally, all terms for finite *s* produce random phases that we can safely neglect ( large) within the approximation. This means practically that all resonances are omitted.

## Appendix E Scattering functions of random media

For many random structures the scattering function developed by Teubner & Strey (1987 ▸) applies quite well. Initially, this was developed for microemulsions and the arguments are based on a functional for the thermodynamic free energy. However, it was found that the scattering functions also work quite well for porous media (Dahl *et al.*, 2024 ▸), possibly because in the production of the material similar arguments hold. Finally, spinodal decomposition at late stages (Cahn, 1965 ▸; Ban *et al.*, 2023 ▸; Skripov & Skripov, 1979 ▸) produces structures related to a peak in the scattering function and a Porod behavior for large *q*. The well known formula for the scattering profile (Endo *et al.*, 2001 ▸) is

Note that  is connected to the fraction of hydrogenous material for neutron scattering, *i.e.* usually to the fraction of one bulk material (oil or water) plus the surfactant. The scattering function is tightly connected to the real-space correlation function  =  [this term would replace the original spherical correlation function of a spherical colloid in equation (18)]. The correlation length ξ describes a finite correlation volume in the sample in which the structure is related to itself. The modulus of the wavevector  describes the preferred distance *d* of alternating domains. Even for  the whole formalism makes sense and was developed by Debye and Büche (Koberstein & Stein, 1980 ▸). For microemulsions, this formalism describes the bulk scattering (*i.e.* the two domains oil and water carry the major contrast). For a heuristic approach to the surface scattering (or the film scattering in microemulsions), we want to develop a relatively simple formula that involves only a minimum set of additional parameters. Usually, the film contrast is related to the square of the original real-space correlation function (Stephenson, 1966 ▸; Roux *et al.*, 1990 ▸; Roux *et al.*, 1992 ▸), *i.e.*. However, the origin of the bulk correlation function is in the middle of a domain, while for the film contrast we need to place the spatial origin onto the film, *i.e.*. This correlation function now describes a divergence for , but it causes a  asymptote at large *q*, which is desired for a surface scattering function. The scattering function is now

Here, the respective composition of hydrogenous material is called . The first term now describes the film scattering (with a thickness δ = 12 Å for the surfactant C_10_E_4_) which is tightly connected to fluctuations of the oil and water domains. The structural volume  connected to this term arises from the patch area . The second term was heuristically added (Nallet *et al.*, 1990 ▸) because the surfactant concentration may also fluctuate on large scales like a two-component Ising critical fluid (without preferred structure) but seems to be invisible under bulk contrast (however, it might be connected to the extra surface of the bulk Porod scattering at higher *q* resulting from short-wavelength fluctuations). While Nallet connected this term to a Lorentz peak centered at *q* = 0, Daicic *et al.* (1995 ▸) connected the amplitude to the osmotic compressibility (Nallet *et al.*, 1990 ▸), *i.e.* =  to . The first proportionality holds for simple microemulsions with three components only, and the latter one for polymer-filled microemulsions, *i.e.* the system then becomes dominated by entropic springs that keep the membranes apart from each other (Endo *et al.*, 2001 ▸). The energy density or pressure Π arises from the arrangement of the membranes with a volume fraction . Note that . This second term of equation (87) of the osmotic compressibility is based on thermodynamics and may be zero for static porous materials. In the classical Debye–Büche approach, the two different terms become indistinguishable anyway.

The strength of this heuristic approach for equation (87) lies in the fact that the structural parameters ξ and *d* are shared between bulk and film contrast and only one additional amplitude  is introduced. Finally, it also has a physical meaning that is connected to the osmotic compressibility.

Now we discuss the quality of equation (87) in the context of measurements (Endo *et al.*, 2001 ▸). We multiplied this expression by a factor  to account for the film roughness. As the structural parameters *d* and ξ are given by the bulk measurements and one only needs to interpolate the values as a function of the membrane volume fraction Ψ, there are only two amplitudes that we treat as free parameters:  and . The results are displayed in Fig. 10 ▸ and the parameters are given in Table 2 ▸. The obtained values for  agree within ±7% with the calculated expectations, thus demonstrating the quality of the new expression.

While in the beginning we focused on real microemulsions which allow us to compare the derived functions with real experiments, the focus now is on porous materials which could have extended pore spaces of *d* ∼ 0.1 to 10 µm. Thus, the description becomes interesting for multiple scattering issues, and so we derive the corresponding scattering functions that were used in the main article [equation (38)] and may apply for porous materials. For the bulk scattering we obtain

The corresponding volume is . For the surface scattering we obtain

As mentioned above, the corresponding area is . The classical Debye–Büche (Koberstein & Stein, 1980 ▸) correlation function is obtained in the limit of . The corresponding correlation function is a single exponential according to . The simpler bulk scattering is now

Here, the volume  applies. For the surface scattering we arrive at the following:

The corresponding area is . With these formulae we have another set of expressions that would allow the modeling of porous media including coherent multiple scattering.

## References

[bb1] Authier, A. (2001). *Dynamical theory of X-ray diffraction*, pp. 534–551. Dordrecht: Springer Netherlands.

[bb2] Ban, M., Woo, D., Hwang, J., Kim, S. & Lee, J. (2023). *Acc. Chem. Res.* **56**, 3428–3440. 10.1021/acs.accounts.3c00524 37964510

[bb3] Barker, J. G., Glinka, C. J., Moyer, J. J., Kim, M. H., Drews, A. R. & Agamalian, M. (2005). *J. Appl. Cryst.* **38**, 1004–1011.

[bb4] Beaucage, G. (1996). *J. Appl. Cryst.* **29**, 134–146.

[bb5] Beck, C., Grimaldo, M., Braun, M. K., Bühl, L., Matsarskaia, O., Jalarvo, N. H., Zhang, F., Roosen-Runge, F., Schreiber, F. & Seydel, T. (2021). *Soft Matter* **17**, 8506–8516. 10.1039/d1sm00418b 34490428

[bb6] Berk, N. & Hardman-rhyne, K. (1986). *Phys. B+C* **136**, 218–222.

[bb7] Cahn, J. W. (1965). *J. Chem. Phys.* **42**, 93–99.

[bb8] Chong, Y. D. (2024). *Quantum mechanics III.* LibreTexts. https://phys.libretexts.org/Bookshelves/Quantum_Mechanics/Quantum_Mechanics_III_(Chong).

[bb9] Cohen-Tannoudji, C., Diu, B. & Laloë, F. (1986). *Quantum mechanics*, Vol. 2. Wiley VCH.

[bb10] Copley, J. R. D. (1988). *J. Appl. Cryst.* **21**, 639–644.

[bb11] Dahl, M., Gommes, C. J., Haverkamp, R., Wood, K., Prévost, S., Schröer, P., Omasta, T., Stank, T. J., Hellweg, T. & Wellert, S. (2024). *RSC Adv.* **14**, 28272–28284. 10.1039/d4ra04090b PMC11372560 39239284

[bb12] Daicic, J., Olsson, U., Wennerström, H., Jerke, G. & Schurtenberger, P. (1995). *Phys. Rev. E* **52**, 3266–3269. 10.1103/physreve.52.3266 9963783

[bb13] Debye, P., Anderson, H. R. & Brumberger, H. (1957). *J. Appl. Phys.* **28**, 679–683.

[bb14] Endo, H., Mihailescu, M., Monkenbusch, M., Allgaier, J., Gompper, G., Richter, D., Jakobs, B., Sottmann, T., Strey, R. & Grillo, I. (2001). *J. Chem. Phys.* **115**, 580–600.

[bb15] Faran, J. J. Jr (1951). *J. Acoust. Soc. Am.* **23**, 405–418.

[bb16] Gibaud, A. (1999). *Specular reflectivity from smooth and rough surfaces*, pp. 87–120. Berlin: Springer.

[bb17] Glatter, O. & Kratky, O. (1982). *Small-angle X-ray scattering.* Academic Press. 10.1016/0076-6879(79)61013-3 481226

[bb18] Gommes, C. J., Zorn, R., Jaksch, S., Frielinghaus, H. & Holderer, O. (2021). *J. Chem. Phys.* **155**, 024121. 10.1063/5.0053446 34266279

[bb19] Hamley, I. W. (2021). *Small-angle scattering: theory, instrumentation, data, and applications.* John Wiley & Sons.

[bb20] Hansen, S. (2000). *J. Appl. Cryst.* **33**, 1415–1421.

[bb22] Hentschel, M. P., Hosemann, R., Lange, A., Uther, B. & Brückner, R. (1987). *Acta Cryst.* A**43**, 506–513.

[bb21] Holderer, O., Landman, J., Kohlbrecher, J., Wu, B., Zolnierczuk, P., Müller, M., Frielinghaus, H., Förster, S., Schwärzer, K., Sagis, L., Shen, P., Yang, J. & Heiden-Hecht, T. (2025). *Adv. Mater. Interfaces* e00368.

[bb23] Jaksch, S., Pipich, V. & Frielinghaus, H. (2021). *J. Appl. Cryst.* **54**, 1580–1593. 10.1107/S1600576721009067 PMC8662966 34963761

[bb24] Jensen, G. V. & Barker, J. G. (2018). *J. Appl. Cryst.* **51**, 1455–1466. 10.1107/S1600576718010816 PMC6157706 30279642

[bb25] Ji, Y., Radlinski, A. P., Blach, T., de Campo, L., Vu, P., Roshan, H. & Regenauer-Lieb, K. (2022). *Fuel* **325**, 124957.

[bb26] Kjems, J., Freltoft, T., Richter, D. & Sinha, S. (1986). *Phys. B+C* **136**, 285–290.

[bb27] Koberstein, J. T. & Stein, R. S. (1980). *J. Polym. Sci. Polym. Phys. Ed.* **18**, 199–205.

[bb28] Koutsioubas, A., Jaksch, S. & Pérez, J. (2016). *J. Appl. Cryst.* **49**, 690–695.

[bb29] Koutsioubas, A. & Pérez, J. (2013). *J. Appl. Cryst.* **46**, 1884–1888.

[bb30] Magerl, A., Lemmel, H., Appel, M., Weisser, M., Kretzer, U. & Zobel, M. (2024). *J. Appl. Cryst.* **57**, 1282–1287. 10.1107/S1600576724007246 PMC11460398 39387068

[bb31] Martin, J. E. (1986). *J. Appl. Cryst.* **19**, 25–27.

[bb32] Mazumder, S. & Sequeira, A. (1992). *Pramana J. Phys.* **38**, 95–159.

[bb33] Mie, G. (1908). *Ann. Phys.* **330**, 377–445.

[bb34] Monkenbusch, M. & Richter, D. (2007). *C. R. Phys.* **8**, 845–864.

[bb35] Munoz, P., Ilavsky, J., Newville, M., Wetter, N. U., Lourenço, R. A., Barbosa de Andrade, M., Martins, T. S., Dipold, J., Freitas, A. Z., Cides da Silva, L. C. & Oliveira, C. L. P. (2023). *J. Appl. Cryst.* **56**, 1692–1706.

[bb36] Nallet, F., Roux, D. & Milner, S. (1990). *J. Phys. Fr.* **51**, 2333–2346.

[bb37] Olaofe, G. O. (1970). *Radio Sci.* **5**, 1351–1360.

[bb38] Pedersen, J. S. (1997). *Adv. Colloid Interface Sci.* **70**, 171–210.

[bb39] Pospelov, G., Van Herck, W., Burle, J., Carmona Loaiza, J. M., Durniak, C., Fisher, J. M., Ganeva, M., Yurov, D. & Wuttke, J. (2020). *J. Appl. Cryst.* **53**, 262–276. 10.1107/S1600576719016789 PMC6998781 32047414

[bb40] Roe, R.-J. (2000). *Methods of X-ray and neutron scattering in polymer science.* Oxford University Press.

[bb41] Roux, D., Cates, M. E., Olsson, U., Ball, R. C., Nallet, F. & Bellocq, A. M. (1990). *Europhys. Lett.* **11**, 229–234.

[bb42] Roux, D., Coulon, C. & Cates, M. E. (1992). *J. Phys. Chem.* **96**, 4174–4187.

[bb43] Schelten, J. & Schmatz, W. (1980). *J. Appl. Cryst.* **13**, 385–390.

[bb44] Schmitt, J., Kjellman, T., Kwaśniewski, P., Meneau, F., Pedersen, J. S., Edler, K. J., Rennie, A. R., Alfredsson, V. & Impéror-Clerc, M. (2016). *Langmuir* **32**, 5162–5172. 10.1021/acs.langmuir.6b00572 27148887

[bb45] Shen, J. & Maradudin, A. A. (1980). *Phys. Rev. B* **22**, 4234–4240.

[bb46] Siddique, T., Balu, R., Mata, J., Dutta, N. K. & Roy Choudhury, N. (2022). *Polymers* **14**, 1980. 10.3390/polym14101980 PMC9147594 35631863

[bb47] Skripov, V. P. & Skripov, A. V. (1979). *Sov. Phys. Usp.* **22**, 389–410.

[bb48] Soraruf, D., Roosen-Runge, F., Grimaldo, M., Zanini, F., Schweins, R., Seydel, T., Zhang, F., Roth, R., Oettel, M. & Schreiber, F. (2014). *Soft Matter* **10**, 894–902. 10.1039/c3sm52447g 24835564

[bb49] Squires, G. L. (1996). *Introduction to the theory of thermal neutron scattering.* Courier Corporation.

[bb50] Stephenson, J. (1966). *J. Math. Phys.* **7**, 1123–1132.

[bb51] Teubner, M. & Strey, R. (1987). *J. Chem. Phys.* **87**, 3195–3200.

[bb52] Tong, D. (2017). *Lectures on topics in quantum mechanics.* Lecture notes at University of Cambridge. https://www.damtp.cam.ac.uk/user/tong/topicsinqm.html.

[bb53] Weiss, R. J. (1951). *Phys. Rev.* **83**, 379–389.

[bb54] Ye, X., Zhang, Q., Feng, C., Ge, H. & Jiao, Z. (1996). *Phys. Rev. B* **54**, 14754–14757. 10.1103/physrevb.54.14754 9985484

[bb55] Zhang, F. & Ilavsky, J. (2010). *Polym. Rev.* **50**, 59–90.

